# Metabolic Landscape and Core Regulatory Network of Monocotyledonous and Dicotyledonous Plants in Drought Response Based on Multi-Omics

**DOI:** 10.3390/plants15020299

**Published:** 2026-01-19

**Authors:** Jianing Zhang, Xiangyu Lin, Shixuan Li, Guo Xu, Xumin Ou, Shouchuang Wang, Ke Zhou, Jun Yang

**Affiliations:** 1 National Key Laboratory for Tropical Crop Breeding, School of Breeding and Multiplication (Sanya Institute of Breeding and Multiplication), Hainan University, Sanya 572025, China; jianing.zhang@hainanu.edu.cn (J.Z.); xiangyu.lin@hainanu.edu.cn (X.L.); shixuan.li@hainanu.edu.cn (S.L.); guo.xu@hainanu.edu.cn (G.X.); xumin.ou@hainanu.edu.cn (X.O.); shouchuang.wang@hainanu.edu.cn (S.W.); 2 National Key Laboratory for Tropical Crop Breeding, College of Tropical Agriculture and Forestry, Hainan University, Sanya 572025, China; 3 Yazhouwan National Laboratory, Sanya 572025, China

**Keywords:** drought stress, phenolamides, plant specialized metabolism, transcriptomics, metabolomics, gene regulatory network

## Abstract

Drought stress severely restricts plant growth and substantially reduces crop productivity. Although drought-response mechanisms have been extensively characterized within individual plant species, the conserved metabolic strategies shared across species remain insufficiently understood. To elucidate both conserved and species-specific metabolic mechanisms underlying drought adaptation, we performed an integrated transcriptomic and metabolomic analysis in rice, maize, and tomato. Profiling of 543 annotated metabolites revealed strikingly divergent baseline metabolic landscapes: tomato leaves were enriched in triglycerides and anthocyanins, whereas maize and rice accumulated higher levels of glycerophospholipids, tricin-derived flavonoids, and B vitamins. Under drought conditions, these differences were further reflected in the distinct sets of differentially accumulated metabolites (DAMs) detected in tomato (121), rice (98), and maize (94). Despite these species-specific signatures, we identified a conserved drought-responsive module consisting of five phenolamides that were consistently induced across all three species. Reconstruction of the associated regulatory network uncovered divergent enzymatic control strategies governing phenolamide biosynthesis: the drought-induced BAHD acyltransferases *OsPHT4* in rice and *SlPHT3* in tomato exhibited broad-spectrum catalytic activities, whereas the maize homolog *ZmPHT4* fulfilled a similar biosynthetic role through constitutive, non-drought-inducible activity. Together, this study provides a comprehensive metabolic framework for plant drought response and demonstrates that extensive species-specific metabolic architectures and transcriptional regulatory divergence coexist beneath a conserved core metabolomic response, offering promising targets for the precise genetic enhancement of crop drought tolerance.

## 1. Introduction

Drought is a major abiotic stress that limits global crop production. It impedes crop growth and development by inhibiting photosynthesis, inducing reactive oxygen species accumulation [1], and disrupting key metabolic pathways, ultimately causing substantial yield losses in major agricultural regions worldwide [2,3]. Although the molecular mechanisms underlying drought tolerance in individual crops have been reported [4,5], drought tolerance is a complex trait governed by multiple interconnected signaling pathways and physiological processes. Considerable interspecific variation in drought responses also exists [6], further complicating efforts to elucidate the underlying mechanisms. To date, comparative studies examining drought-response mechanisms across species remain limited [7]. Thus, cross-species comparative analyses are essential for identifying conserved mechanisms, which have great potential for advancing crop drought tolerance and enhancing global food security.

Over the course of evolution, plants have developed sophisticated and multilayered mechanisms to mitigate drought-induced damage, encompassing processes from stress perception and transcriptional regulation to extensive metabolic reprogramming [8]. As direct participants in plant stress responses, changes in the abundance and composition of metabolites serve as sensitive indicators of a plant’s internal adaptive strategies [9]. For instance, primary metabolites such as proline, trehalose, and unsaturated fatty acids accumulate under drought stress [10,11], functioning as osmoregulators and signaling molecules to maintain cellular osmotic homeostasis and alleviate stress-induced damage. Meanwhile, secondary metabolites, including phenolic compounds and terpenoids, enhance plant resistance to oxidative damage by scavenging excess reactive oxygen species, either through their intrinsic antioxidant activities or via activation of antioxidant enzyme systems such as peroxidases and superoxide dismutases [12]. Phenolamides are a class of secondary metabolites formed by the conjugation of hydroxycinnamic acids with polyamines or aromatic amines, and they play a pivotal role in plant defense and stress tolerance [13]. Due to their strong antioxidant, antimicrobial, and cell wall-fortifying activities, they directly enhance plant fitness and resilience under adverse environmental conditions [14]. Their biosynthesis is primarily mediated by two major acyltransferase families: members of the BAHD family catalyze the acylation of aliphatic polyamines, while the tyramine hydroxycinnamoyltransferases (THTs) of the GNAT family specifically acylates aromatic amines, collectively contributing to the structural diversity of phenolamides [15,16]. Phytohormones play a key role during stress responses by orchestrating signal perception, defense systems, and growth regulation [17]. Recent studies have revealed extensive crosstalk between phenolamide accumulation and hormonal signaling upon stress. Hormones such as jasmonic acid and abscisic acid, in coordination with transcription factors like MYBs and bZIPs, regulate the expression of BAHD and GNAT family genes, thereby promoting the accumulation of diverse phenolamides [18,19,20]. For instance, in tomato, the transcription factor *SlMYB13* positively regulates the phenolamide biosynthetic gene clusters BGC7 and BGC11, promoting the accumulation of ABA and various phenolamides, which collectively strengthen drought resistance [21]. Plant responses to drought stress are dynamic, yet changes in metabolite accumulation and associated gene activation during the drought response remain inadequately characterized.

Drought stress trigger extensive transcriptional reprogramming in plants, thereby regulating modulating metabolite biosynthesis and turnover. Integrated transcriptomic and metabolomic approaches provide powerful tools for elucidating the interplay between gene regulation and metabolic pathways under drought stress. For example, in maize, melatonin has been shown to enhance drought tolerance by promoting root growth and activating the expression of genes involved in hormone signaling and the phenylpropanoid biosynthesis pathway [22]. In rice, overexpression of *OsASR5* confers drought tolerance by elevating endogenous abscisic acid levels and modulating hydrogen peroxide signaling, ultimately reducing stomatal conductance [23]. Distinct stress-responsive strategies have also evolved between dicots and monocots. In dicotyledonous plants, specific ATG8-interacting proteins (ATI3 and UBAC2) play important roles in disease resistance and thermotolerance by mediating selective autophagy [24]. In monocotyledonous plants such as rice, a conserved hydroxycinnamoylputrescine (HP) biosynthetic gene cluster contributes to enhanced disease resistance through HP accumulation [25]. Although numerous drought-response mechanisms have been identified independently in monocot and dicot crops, systematic cross-species comparisons of conserved versus specific regulatory strategies between monocot and dicot crops remain scarce.

In this study, we selected maize (B73), rice (ZH11), and tomato (MicroTom) as representative model crops for monocots (maize and rice) and dicots (tomato). Using comparative transcriptomic and metabolomic analyses, we first characterized drought-induced metabolic dynamics in each species and then identified conserved responses across the three crops. Our integrated multi-omics approach revealed that although drought stress reshaped the metabolomes of maize, rice, and tomato in predominantly species-specific ways, it consistently upregulated five key phenolamide compounds in all three species. Further multi-omics integration delineated a core regulatory network underlying phenolamide biosynthesis and pinpointed critical BAHD acyltransferase genes within this pathway. Functional characterization uncovered divergent enzymatic strategies among species: *SlPHT3* and *OsPHT4* exhibited broad substrate promiscuity and were strongly induced by drought, whereas the maize homolog *ZmPHT4* maintained constitutive catalytic activity but was not drought-inducible, reflecting divergence at both transcriptional and enzymatic levels. Together, our findings highlight the diversity of metabolites and gene regulatory networks underlying plant drought responses and provide a conceptual framework for identifying key drought-resilience metabolites and genes across crop species.

## 2. Results

### 2.1. Monocot and Dicot Crops Have Different Metabolic Profiles

**Figure 1:**
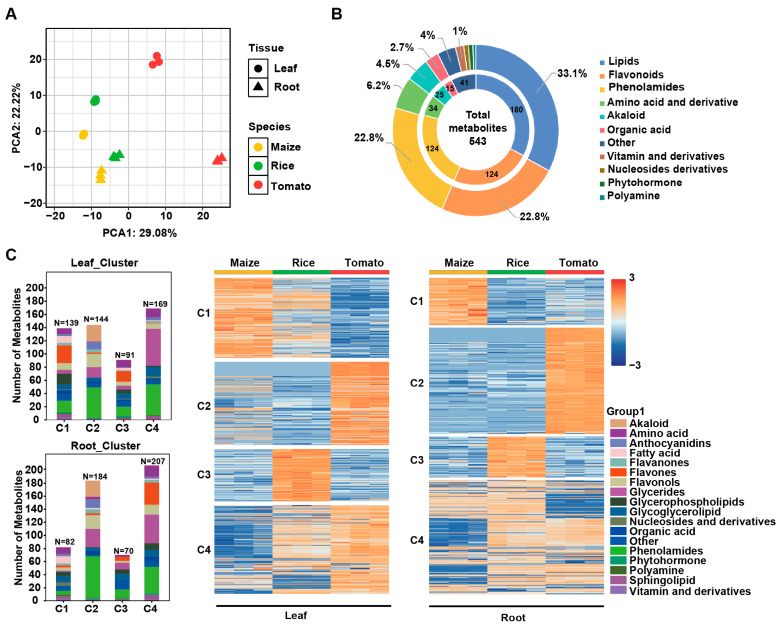
Comparative analysis of baseline metabolic profiles in maize, rice, and tomato. (**A**) Principal component analysis (PCA) based on total ion chromatogram (TIC) data obtained from leaf (circles) and root (triangles) tissues of the three crop species, with the x- and y-axes representing the scores of principal component 1 (PC1) and principal component 2 (PC2), respectively. (**B**) Annotation and relative composition of metabolites identified across all samples. A total of 543 metabolites were identified in leaf and root tissues of the three crop species in this study. (**C**) Clustered heatmap analysis of targeted metabolomics data. The left panels show stacked bar charts indicating the number of metabolites (N) in each cluster (C1–C4) in leaf and root tissues, with different colors representing different metabolite classes, while the right panels display heatmaps illustrating the relative abundance of metabolites across species and tissues (red, higher; blue, lower).

To systematically delineate the basic metabolic diversity between monocotyledonous and dicotyledonous crops, we first constructed a baseline metabolic landscape for three species (tomato, rice, and maize) under normal growth conditions using LC-TOF-MS. By applying an untargeted metabolomics approach, we identified 4995 metabolic signals across the three crop species. Significant differences in the number and abundance of metabolic features displayed in the total ion chromatograms were observed across tissues and species (Figure S1A). We performed a principal component analysis (PCA) on the non-targeted metabolomic data for all samples to further examine differences among tissues and species. Principal components 1 and 2 explained 29.08% and 22.22% of the total variance, respectively (Figure 1A). PCA results showed that metabolic profiles of the same tissue from the same species were tightly clustered, indicating good reproducibility among samples. More importantly, clear separation was observed between dicot tomato and monocot rice or maize samples. Moreover, root and leaf samples from the same species were also distinctly separated from each other. These distribution patterns clearly demonstrate significant differences in metabolic profiles between different species and tissues.

To gain deeper insights into the metabolic diversity of the three species, we characterized metabolites based on their fragmentation pattern, mass-to-charge ratio (*m*/*z*), neutral loss type, and retention time (RT) (Figure S1B). Based on these features, we annotated a total of 543 metabolites by cross-referencing data from the literature and databases such as METLIN. The metabolites included 180 lipids, 124 flavonoids, 124 phenolamides, 34 amino acids and their derivatives, 25 alkaloids, 15 organic acids, six vitamins and their derivatives, five nucleotides and their derivatives, five plant hormones, three polyamines, and 22 other unclassified compounds (Figure 1B; Table S1). We quantified these metabolites using dynamic multiple reaction monitoring (dMRM).

A clustering heatmap based on the targeted metabolomics data clearly illustrates the distribution of differentially accumulated metabolites (DAMs) across the three crop species (Figure 1C). Triglycerides were enriched in tomato, whereas rice and maize were higher in glycerolipids and glycerophospholipids. Maize had higher levels of multiple polyunsaturated fatty acids (α-linolenic acid, linoleic acid, and palmitoleic acid), indicating marked differences in lipid metabolism among species (Figure 1C; Figure S2A). In terms of flavonoids, certain anthocyanins were more abundant in tomato, such as cyanidin, delphinidin, and petunidin glycosides, whereas maize and rice preferentially accumulated tricin and its glycosides, as well as multiple flavonoid glycosides (e.g., apigenin and luteolin) (Figure 1C; Figure S2B). Furthermore, B vitamins (niacin, pantothenic acid, and riboflavin) were generally more abundant in maize and rice than in tomato. These vitamins possess antioxidant and free radical scavenging potential [26], potentially increasing plant resistance to abiotic stress. Phenolamides represent crucial stress-responsive metabolites in plants [27]. The accumulation of phenolamide precursors (polyamines) exhibited marked species differences. Tyramine levels were significantly higher in tomato than maize or rice tissues, while maize and rice leaves accumulated higher levels of putrescine and spermidine (Figure S2C). As a result, the accumulation of downstream phenolamide products exhibited similar species-specific patterns. Notably, the abundance of amino acid precursors to polyamine synthesis was negatively correlated with that of the polyamines themselves (Figure S2D). The described differences in metabolite dynamics among species likely reflect species-specific metabolic pathway regulation and physiological adaptation strategies.

### 2.2. Construction of the Drought Responsive Metabolic Landscape Identifies Conserved Phenolamides Accumulation

**Figure 2:**
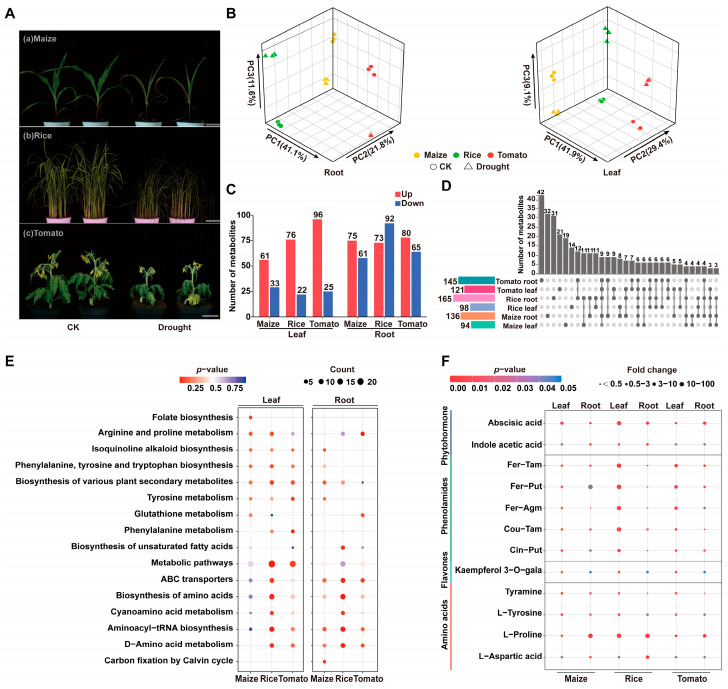
Metabolomic responses of maize, rice, and tomato to drought stress. (**A**) Phenotypic analysis of the three crop species after 10 days of natural drought treatment. Scale bar = 5 cm. (**B**) Principal component analysis (PCA) of targeted metabolites in leaf and root tissues of the three crop species under control (CK) and drought conditions, where PC1, PC2, and PC3 represent the first, second, and third principal components, respectively. (**C**) Comparative analysis of upregulated and downregulated differentially accumulated metabolites (DAMs) across species and tissues following drought treatment, with red indicating upregulated metabolites and blue indicating downregulated metabolites. (**D**) UpSet plot illustrating the overlap of DAMs among different crop species and tissues. (**E**) KEGG pathway enrichment analysis of drought-induced DAMs in leaf and root tissues. (**F**) Bubble plot showing core metabolites responsive to drought stress across species and tissues.

To construct a drought-responsive metabolic landscape and uncover conserved adaptive mechanisms across monocot and dicot species, we performed LC-MS/MS-based targeted metabolomic analysis on tomato, rice, and maize subjected to 10 days of drought treatment. Phenotypic observations revealed species-specific symptoms of stress: tomato seedlings showed significantly inhibited growth with stunted stature and leaf yellowing, while rice and maize leaves exhibited marked wilting and curling (Figure 2A). A PCA of the targeted metabolomics data revealed substantial shifts in metabolite abundance before and after drought stress (Figure 2B). Compared to the controls, 121, 98, and 94 DAMs were identified in the leaves of drought-stressed tomato, rice, and maize plants, respectively, while 145, 165, and 136 DAMs were identified in drought-stressed tomato, rice, and maize roots (Figure 2C). To compare metabolite accumulation patterns, we used an UpSet plot to visualize the number of shared DAMs across species and tissues (Figure 2D), finding substantial differences for most metabolites, potentially reflecting interspecific variation in drought tolerance.

To gain deeper insights into the metabolic responses to drought stress, we performed KEGG pathway enrichment analysis on the drought-responsive differentially accumulated metabolites (DAMs). Figure 2E illustrates representative biological processes, providing a detailed comparison of the metabolic responses to drought across the three species. In leaves, pathways such as “Biosynthesis of various plant secondary metabolites” and “Biosynthesis of phenylalanine, tyrosine, and tryptophan” were commonly enriched. Notably, in the upstream metabolic pathways of phenolamide synthesis, tomato exhibited significant enrichment in “Phenylalanine metabolism” and “Tyrosine metabolism”, whereas maize and rice showed more pronounced enrichment in “Arginine and proline metabolism”. The latter pathway was also significantly enriched in tomato roots, revealing a distinct metabolic pattern compared to the two monocot species. Furthermore, “Folate biosynthesis” was specifically enriched in maize leaves. In roots, the co-enrichment of “ABC transporters”, “Aminoacyl-tRNA biosynthesis”, and “Biosynthesis of amino acids” reflects a common response mechanism involving stress signal transduction and enhanced amino acid metabolism across all three species. Rice exhibited significant enrichment of these pathways in both the leaves and roots. These species-specific metabolic changes highlight the divergent strategies employed by different plant lineages to cope with drought stress.

Furthermore, we identified both conserved (shared across species) and species-specific drought-responsive DAMs. Under drought stress, the most significantly upregulated metabolites included phenolamides, lipids, amino acids, and their derivatives. Some drought-related DAMs exhibited distinct accumulation patterns in leaf versus root tissues, likely reflecting tissue-specific adaptive responses. Analysis of species-specific DAMs revealed distinct profiles for each species. Tomato unique DAMs included anthocyanins and triglycerides that accumulated in leaves. Rice unique DAMs primarily consisted of amino acids and their derivatives, with fatty acids and glycerophospholipids also being upregulated in root tissues. Maize-specific DAMs included flavanols and membrane lipid metabolites. Cross-species comparison revealed that B vitamins accumulated in both maize roots and tomato leaves, while sphingolipids and plant hormones were upregulated in leaves of the two monocot species but significantly downregulated in tomato. Notably, several polyamines, including putrescine, spermidine, and spermine, exhibited completely opposite accumulation trends in maize and rice compared to tomato (Figure S3).

We further focused on the DAMs showing conserved responses across species. This analysis led to the identification of five key phenolamides (cinnamoyl putrescine [Cin-Put], coumaroyl tyramine [Cou-Tam], feruloyl agmatine [Fer-Agm], feruloyl putrescine [Fer-Put], and feruloyl tyramine [Fer-Tam]), two amino acids and their derivatives (tyramine and tyrosine), and a single flavonoid (kaempferol 3-*O*-galactoside). Under drought stress, abscisic acid and proline accumulated in all three crop species. Proline, which functions as an osmotic regulator, was simultaneously upregulated in the roots of all three species, while abscisic acid accumulated in all tissues examined (Figure 2E). Interestingly, the conserved DAMs included lipid molecules enriched with C18 fatty acid chains, suggesting a pivotal role of C18 fatty acids in drought tolerance (Table S2). These metabolite categories are implicated in the drought responses of all three species and are known to be generally crucial for plant stress responses. This underscores the importance of conserved stress-response metabolites and provides key metabolic pathways and signaling clues for understanding crop adaptation mechanisms to drought stress.

### 2.3. Monocot and Dicot Crops Show Distinct Transcriptomic Alterations in Response to Drought

In the previous metabolomic analysis, we found that under drought stress, phenolamide metabolites exhibited a significant co-response pattern in the leaves of the three crop species. To better understand how drought stress affected our three focal crop species, we performed transcriptome sequencing on 18 cDNA libraries (i.e., for three species, two treatments, and three biological replicates) derived from crop leaf tissues. Following quality control, we obtained a total of 150 Gb of clean data, with each sample yielding over 7.5 Gb of clean data. Both Q20 and Q30 base coverage exceeded 98%, with the GC content ranging between 40% and 60%. We were able to successfully map between 98.25% and 99.08% of clean reads to the reference genome, confirming that the sequence data quality was suitable for our analyses.

**Figure 3:**
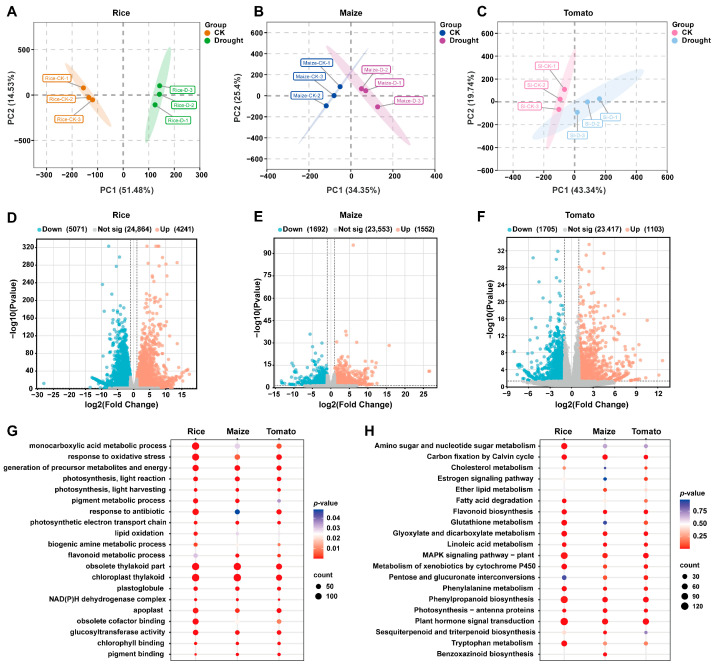
Transcriptome analysis of rice, maize, and tomato seedlings under drought stress. (**A**–**C**) Principal component analysis (PCA) of rice (**A**), maize (**B**), and tomato (**C**) seedlings under drought (Drought) and control (CK) conditions, where PC1 and PC2 represent the first and second principal components, respectively. (**D**–**F**) Volcano plots showing differentially expressed genes (DEGs) in rice (**D**), maize (**E**), and tomato (**F**); blue, orange, and grey dots represent downregulated, upregulated, and non-significant genes, respectively. (**G**) Gene Ontology (GO) enrichment analysis of DEGs in each species, with point size indicating the number of enriched genes and color representing significance (*p* value), where deeper red indicates higher statistical significance. (**H**) KEGG pathway enrichment analysis of DEGs in each species, with point size indicating the number of enriched genes and color representing significance (*p* value), where deeper red indicates higher statistical significance.

In the transcriptome-based PCA plots for each species, drought-stressed samples were well-separated from control samples (Figure 3A–C; Table S4). This finding aligns closely with patterns observed for the metabolome-based PCA (Figure 1A) and indicates that drought stress also influenced gene expression. We identified differentially expressed genes (DEGs) between the drought and control treatments as those exhibiting a log2 fold change ≥ 1 and *p* < 0.05 (Figure 3D–F). A total of 9312 DEGs were identified in rice (4241 upregulated and 5071 downregulated), 3244 in maize (1552 upregulated and 1692 downregulated), and 2808 in tomato (1103 upregulated and 1705 downregulated).

To connect the DEGs to associated metabolic pathways, we conducted GO and KEGG enrichment analyses. The GO enrichment analysis (Figure 3G) revealed that multiple biological functions associated with plant stress responses were significantly enriched across all three species. These primarily encompassed biogenic amine metabolism, flavonoid metabolic processes, lipid oxidation, and responses to oxidative stress. Similarly, the KEGG pathway analysis (Figure 3H) revealed pathways that were enriched across all three species, including flavonoid biosynthesis, phenylpropanoid metabolism, and plant hormone signal transduction pathways. Additionally, we observed species-specific enrichment patterns for each crop. Metabolic pathways uniquely enriched in maize included benzoxazinoid biosynthesis, ether lipid metabolism, and sesquiterpenoid and triterpenoid biosynthesis, while pathways uniquely enriched in rice included amino sugar and nucleotide sugar metabolism, fatty acid degradation, glutathione metabolism, and tryptophan metabolism. Metabolic pathways uniquely enriched in tomato included cholesterol metabolism, estrogen signaling, and pentose and glucuronic acid conversion. Similarly, the KEGG pathway analysis identified multiple secondary metabolite synthesis pathways that were enriched in both the monocot and dicot crops. These metabolic pathways are thus likely crucial to drought stress responses in both monocot and dicot crops.

### 2.4. Integrated Multi-Omics Analysis Constructs a Core Regulatory Network for Conserved Phenolamide Biosynthesis

**Figure 4:**
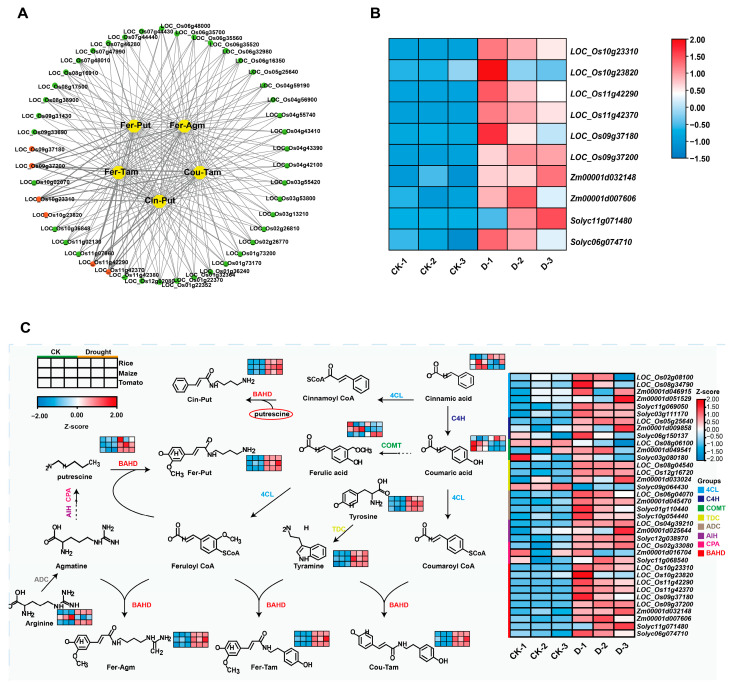
Gene–metabolite correlation networks and phenolamide accumulation changes under drought stress in rice, maize, and tomato. (**A**) Gene–metabolite correlation network in rice. Yellow nodes represent metabolites, green nodes represent genes, and red nodes represent the BAHD acyltransferase family members. Lines indicate significant correlations (|r| > 0.8, *p* < 0.05). (**B**) Gene expression heatmap for BAHD acyltransferase family members, which are induced by drought. The color gradient represents changes in relative expression (log2-transformed values), with blue indicating downregulation and red indicating upregulation. (**C**) Heatmap for gene expression and metabolite accumulation in the phenolamide biosynthesis pathway. Red and blue represent increased and decreased levels, respectively. Key enzymes include 4-coumarate-CoA ligase (4CL), arginine decarboxylase (ADC), agmatine iminohydrolase (AIH), BAHD acyltransferase family (BAHD), coumarate-3-hydroxylase (C3H), cinnamate-4-hydroxylase (C4H), caffeic acid O-methyltransferase (COMT), N-carbamoylputrescine amidohydrolase (CPA), and tyrosine decarboxylase (TDC). Related phenolamides include feruloylagmatine (Fer-Agm), feruloyltyramine (Fer-Tam), coumaroyltyramine (Cou-Tam), feruloylputrescine (Fer-Put), and cinnamoylputrescine (Cin-Put).

As we observed significant phenolamide accumulation under drought stress, we explored potential associations between the drought-related DEGs and five key phenolamides (Cin-Put, Cou-Tam, Fer-Agm, Fer-Put, and Fer-Tam) that were upregulated by drought in all three crops. Our goal was to elucidate common drought response mechanisms shared by monocots and dicots (Figure 4A; Figure S4). As phenolamide synthesis is primarily regulated by the BAHD acyltransferase family (located downstream of the phenylpropanoid pathway), we identified BAHD family genes highly correlated with the abundance of the five key phenolamides (*p* < 0.05, PCC > 0.8) (Table S4). Six BAHD family genes were identified in rice (LOC_Os09g37180, LOC_Os09g37200, LOC_Os10g23310, LOC_Os10g23820, LOC_Os11g42290, and LOC_Os11g42370), two in maize (Zm00001d007606 and Zm00001d032148), and two in tomato (Solyc06g074710 and Solyc11g071480), all of which were significantly upregulated under drought conditions (Figure 4B). Notably, upregulation of *OsPHT4* (LOC_Os09g37200) and *SlAT1.2* (Solyc11g071480) has been shown to enhance drought tolerance [21,28], validating the reliability of our screening approach and suggesting a key role for phenolamide biosynthesis in mediating drought resistance.

To further explore the role of the five key phenolamides in drought tolerance, we documented the synthesis of these phenolamides across our focal crop species (Figure 4C). Under drought stress, precursor substances for amine synthesis increased in abundance in all three crops, suggesting that adequate substrate supply may be a crucial prerequisite for enhanced phenolamide synthesis. Concurrently, the expression of key genes in the phenylpropanoid pathway (i.e., 4-coumarate: CoA ligase [4CL], arginine decarboxylase [ADC], and cinnamate 4-hydroxylase [C4H]) was broadly upregulated, indicating the coordinated activation of this pathway during the drought response. Although we found differences among the focal crops in terms of phenolamide synthesis-related genes and metabolite accumulation, our multi-omics data consistently showed that drought stress promoted phenolamide biosynthesis. These findings confirm the crucial role of phenolamide accumulation in plant responses to drought stress.

### 2.5. Functional Validation Reveals Divergent Transcriptional Regulation of Catalytic Enzymes Within Monocots

**Figure 5:**
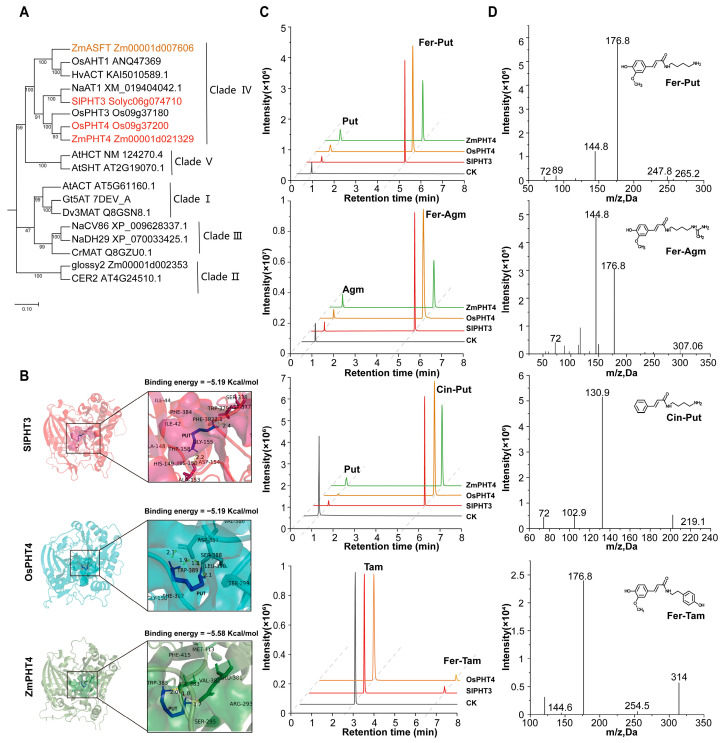
Phylogenetic analysis and enzymatic activity validation of candidate BAHD acyltransferases from maize, rice, and tomato. (**A**) Phylogenetic tree showing the relationships between candidate BAHD acyltransferase genes and previously characterized BAHD acyltransferases. (**B**) Molecular docking analysis of three candidate BAHD proteins with putrescine (Put) as the substrate; right panels show enlarged views of the ligand-binding sites and the corresponding binding energy values, with putrescine highlighted in blue. (**C**) In vitro enzyme assays of homologous BAHD proteins from maize, rice, and tomato using agmatine (Agm), putrescine (Put), and tyramine (Tam) as substrates; representative chromatograms of the reaction products are shown. (**D**) Mass spectra confirming the enzymatic products shown in panel (**C**).

To functionally characterize the key BAHD acyltransferases implicated by our core regulatory network, we performed a phylogenetic analysis alongside previously reported enzymes with confirmed activity [29] (Table S6). The analysis showed that *SlPHT3* (Solyc06g074710), *OsPHT4* (LOC_Os09g37200), and *ZmASFT* (Zm00001d007606) were clustered in Clade IV and showed a close phylogenetic relationship with the known transferases that catalyze putrescine (Put), cadaverine (Tam), and agmatine (Agm), suggesting that they may exert similar biological functions (Figure 5A).

We performed molecular docking [30] of the three proteins and found that their active pockets largely overlapped. All three substrates (i.e., Agm, Put, and Tam) could embed into the active pocket and interact with key residues such as ASP and HIS, ensuring binding stability and providing a structural basis for the catalytic reaction. This may confer broad-spectrum catalytic capability to these enzymes (Figure 5B; Figure S5). Although molecular docking was also simulated for the homologous protein *ZmASFT* (Zm00001d007606) in maize, the minimum binding energies for Agm and Put were relatively high, suggesting that it may not be involved in catalyzing the formation of the corresponding products (Table S7).

To validate the functions of these enzymes, we purified recombinant proteins with glutathione-S-transferase (GST) tags and conducted in vitro enzymatic assays using Agm, Put, and Tam as substrates and cinnamoyl-CoA (Cin-CoA), feruloyl-CoA (Fer-CoA), and p-coumaroyl-CoA (Cou-CoA) as acyl donors. Using LC-MS, we found that SlPHT3-GST and OsPHT4-GST were able to catalyze all three substrates, producing Cin-Put, Fer-Agm, Fer-Put, and Fer-Tam. In contrast, the protein encoded by *ZmASFT* did not catalyze any of the substrates (Figure 5C,D). To search for an enzyme in maize that shared the same function as OsPHT4 and SlPHT3, we compared protein sequences (Figure S6) and identified *ZmPHT4* (Zm00001d021329) as highly homologous to OsPHT4 and SlPHT3. Subsequent enzyme activity assays confirmed that ZmPHT4-GST could catalyze Agm and Put but not Tam.

In summary, our functional validation confirms the catalytic roles of key acyltransferases in the phenolamide biosynthesis network. Crucially, it reveals a divergent transcriptional regulation strategy for these enzymes within monocots: while the catalytic functions in rice and tomato are performed by drought-induced enzymes (*OsPHT4* and *SlPHT3*), in maize, the same biochemical role is fulfilled by *ZmPHT4*, which is not drought-induced but possesses constitutive catalytic activity.

## 3. Discussion

Drought stress represents a critical abiotic constraint limiting the biomass and yield of crop plants worldwide [6]. Characterizing conserved metabolic pathways that underlie plant stress resistance can offer novel strategies for crop improvement, especially in the face of the extreme environmental pressures linked to global change. Studies of major crops such as maize and rice have revealed that species-specific benzoxazinoids, phenylpropanoids, and terpenoids mediate plant responses to adverse environmental conditions [31,32,33]. However, our understanding of conserved metabolic responses underlying drought resistance remains limited, making it difficult to identify potential mechanisms operating across species. By integrating comparative metabolomic and transcriptomic analyses for the dicot model plant tomato and the monocot model plants rice and maize, we uncovered drought-induced shifts in metabolite abundance and composition, including shifts unique to a single crop and those shared among crops. These results indicate that crop adaptation to drought stress does not rely solely on crop-specific metabolic regulatory modes, but instead arises from the integration of species-specific metabolic allocation strategies with conserved defensive metabolic modules shared across species. We also identified key candidate genes and phenolamides that may contribute to drought adaptation in these crop species.

Our findings reveal evolutionary divergences between monocotyledons and dicotyledons in how they energize their inherent defense systems. Whilst polyamines represent a shared response product, monocotyledons rely on the arginine/proline pathway whereas dicotyledons depend on the phenylalanine/tyrosine pathway, indicating profound differences in resource allocation. Monocotyledons may have evolved mechanisms to more efficiently convert nitrogen into polyamines, which also serve osmoregulatory functions. In contrast, tomatoes rely more heavily on the phenylalanine branch, which integrates drought defense with their characteristic flavonoid (anthocyanin) metabolism. This divergence reflects how distinct plant lineages reconfigure fundamental metabolic pathways to support convergent functional outputs. In addition, differences in drought tolerance among crops should be interpreted in the context of their inherent baseline drought resistance. Under drought stress, maize exhibited lower response fold changes in several metabolites known to mitigate drought stress (such as proline and feruloylputrescine), as well as in the phenolamides proposed in this study, compared with rice and tomato (Figure S7). This pattern likely reflects the stronger baseline drought tolerance of maize, which requires only moderate metabolic adjustment under drought conditions. In contrast, rice and tomato, particularly rice as a relatively drought-sensitive crop, rely more heavily on substantial metabolite accumulation to cope with drought stress, resulting in more pronounced metabolic responses. These differences highlight the distinct metabolic strategies adopted by the three crops during drought adaptation.

Phenolamides exhibited consistent drought-induced accumulation across different crop species; however, they are not the sole determinants of crop drought tolerance. Instead, phenolamides are more likely to function as a conserved metabolic hub embedded within a broader drought-responsive metabolic network, acting in concert with amino acids, flavonoids, lipid metabolites, and plant hormone signaling pathways. For example, plant hormones and phenolamides may act synergistically in mediating plant responses to both abiotic and biotic stresses. In this study, under drought stress, both plant hormone signal transduction pathways and the MAPK signaling pathway were significantly upregulated. The functions of these signaling pathways in crop drought tolerance have been extensively validated by numerous studies [34]. Under drought conditions, abscisic acid (ABA) accumulates rapidly and acts as a core regulator of the plant drought response [35]. Phenolamide accumulation can induce ABA accumulation and the expression of key ABA-related genes (such as *ABA3* and *NCED3*), thereby enhancing drought tolerance [21]. This suggests that phenolamides may possess a synergistic or cross-regulatory relationship with the ABA signaling pathway, promoting the accumulation of metabolites with antioxidant activity and activating the ABA metabolic pathway. Interactions between plant hormones and phenolamides in response to drought stress requires further in-depth investigation in crop species.

In this study, drought significantly increased the abundance of various phenolamides, with several phenolamides in the leaves showing significant upregulation. These phenolamides have been reported to play key roles in plant stress responses. For example, feruloyltyramine exhibits direct antimicrobial activity by serving as a structural component of the cell wall, enhancing its strength and restricting pathogen spread, thereby contributing to plant disease resistance [36]. Meanwhile, feruloylputrescine, a drought-responsive phenolamide compound, plays a protective role by helping plants cope with short-term drought [21]. In the roots, only benzoyl cadaverine showed upregulation across all three species. Although its function under drought stress has not been clearly reported, this compound may be associated with the plant’s response to drought stress. Consistent with metabolomic findings, most genes related to phenylpropanoid biosynthesis and amine metabolism, including 4CL, ADC, AIH, and TDC, were upregulated under drought stress. Our transcriptomic and metabolomic results suggest that activation of the phenolamide biosynthetic pathway may represent a conserved plant response to drought stress. In addition, we identified key genes involved in phenolamide synthesis, including Solyc06g074710, LOC_Os09g37200, and Zm00001d021329. The differential regulation of acyltransferases across different species observed in this study may reflect the divergent adaptive strategies of plants in response to drought stress, specifically in their regulation of defense-related secondary metabolic pathways. Defense-related secondary metabolic pathways can exhibit either induced or constitutive regulation in different plants, and such differences are often associated with distinct regulatory strategies for drought stress response. Specifically, the acyltransferase *ZmPHT4,* related to phenolamide biosynthesis in maize, did not show significant induction under drought stress, displaying a constitutive regulation pattern. In contrast, the corresponding enzymes *OsPHT4* in rice and *SlPHT3* in tomato were primarily induced under drought stress, showing a typical induced regulation pattern. These differences suggest that the regulation of acyltransferases may play a role in the formation of different drought tolerance strategies in crops, the catalytic functions of these acyltransferases and their association with phenolamide synthesis have been initially validated. However, the in planta functions of these genes in intact plants require further confirmation through subsequent gene editing experiments (e.g., CRISPR/Cas9-mediated knockout or overexpression). Future studies will focus on verifying the in planta functions of the target genes to clarify their regulatory effects on phenolamide accumulation and crop drought tolerance.

The metabolic changes analyzed in this study were observed after a continuous 10-day drought treatment, a time scale that more likely reflects adaptive metabolic reprogramming formed under prolonged water limitation rather than the rapid responses associated with the initial phase of drought stress. After 10 days of drought stress, in addition to phenolamides, most amino acids and their derivatives, flavonoids, and lipid metabolites also significantly increased in abundance. These key metabolites regulate stress signal transduction, enhancing reactive oxygen species scavenging capacity and improving plant stress tolerance [37,38]. In plant roots, proline accumulation contributes to delays in plant water loss [39], thereby increasing survival and metabolic stability. Here, amino acid accumulation was particularly notable in rice, where γ-aminobutyric acid (GABA) has been demonstrated to enhance antioxidant capacity by upregulating the activity of antioxidant enzymes [40]. Drought also induced the biosynthesis of benzoxazinoids in maize, which are known to alleviate ROS toxicity under drought stress [38]. Flavonoids, as important non enzymatic antioxidants in plants, effectively scavenge reactive oxygen species [41]. Ultraviolet radiation induces flavonoid accumulation, thereby improving plant tolerance to UV-B stress [42]. In rice, overexpression of *OsCHI3* under drought stress promotes flavonoid accumulation, enhances antioxidant enzyme activity, and regulates ABA biosynthesis; collectively, these effects positively impact drought tolerance [43]. Here, drought led to the upregulation of the flavonoid biosynthetic pathway across all three focal crops, underscoring the importance of flavonoids in crop drought responses. In summary, the metabolites identified here collectively constitute the foundation of the plant drought stress response.

Taken together, crop adaptation to drought stress relies on the combined action of conserved metabolic responses and species-specific regulatory strategies, with phenolamides functioning as a conserved metabolic hub embedded within a drought-responsive metabolic network composed of multiple protective metabolites and signaling pathways. Our findings underscore the importance of phenolamides for crop drought tolerance. In this study, we also identified a set of key metabolites and associated candidate genes and verified the catalytic functions of these enzymes in vitro. However, the specific mechanisms by which phenolamides mediate drought tolerance across crop species remain to be fully elucidated. Future studies should focus on characterizing the mechanisms underlying plant drought tolerance as such steps will be critical to both design drought resistant crop varieties and obtain genetic resources for their development.

## 4. Materials and Methods

### 4.1. Plant Materials

Three important crop genotypes were selected as experimental materials, including maize (B73), rice (ZH11), and tomato (MicroTom). All materials were sown and grown in the greenhouse of Sanya Nanfan Research Institute, Hainan University, until reaching the four-leaf-one-heart seedling stage. The experimental group was subjected to drought stress through 10 days of natural drought, whereas the control group received regular irrigation throughout the experimental period. Following the 10-day treatment, leaves and roots were harvested from both experimental and control plants of the three species and immediately placed into 50 mL centrifuge tubes. For each species, three biological replicates (with uniform growth phenotypes) were set up, and all collected samples were rapidly frozen in liquid nitrogen followed by freeze-drying for subsequent analyses.

### 4.2. Preparation of Metabolic Samples

Freeze-dried plant samples were pulverized with a ball mill (MM 400; Retsch, Haan, Germany) at 30 Hz for 1 min to ensure homogeneous powdering. Exactly 100 mg of homogenized sample powder was accurately weighed and transferred into a 2 mL sterile centrifuge tube. Subsequently, pre-cooled (4 °C) 70% (*v*/*v*) methanol (extraction solvent) was added at a 1:10 (*w*/*v*) solid-to-liquid ratio (100 mg powder:1 mL solvent). The mixture was vigorously vortexed for 1 min for thorough mixing, then incubated on ice for 10 min; this cycle was repeated twice more (total 3 cycles) to enhance metabolite leaching. After the final vortex, samples were statically extracted at 4 °C for 12 h in the dark to prevent the photodegradation of light-sensitive metabolites. Post-extraction, mixtures were centrifuged at 12,000× *g* for 10 min (4 °C) to pellet insoluble debris. The supernatant was carefully filtered through a 0.2 μm organic phase syringe filter into a glass autosampler vial, and filtrates were stored at 4 °C until UHPLC-MS analysis.

### 4.3. Metabolite Analysis

Metabolic extracts were subjected to chromatographic separation using an Agilent 1290 ultra-high performance liquid chromatography (UHPLC) system (Santa Clara, CA, USA). Chromatographic separation was achieved on a Waters ACQUITY UPLC HSS T3 column (1.8 μm pore size, 2.1 × 100 mm; Waters, Milford, MA, USA). The mobile phase consisted of solvent A (0.1% formic acid in water, *v*/*v*) and solvent B (0.1% formic acid in acetonitrile, *v*/*v*), with the following gradient elution program: 0 min, 100% A; 2 min, 95% A/5% B; 12 min, 5% A/95% B; 13 min, 5% A/95% B; 13.1 min, 100% A; 17 min, 100% A. The column temperature was maintained at 40 °C, with a constant flow rate of 0.4 mL/min and an injection volume of 2 μL. Eluted metabolites were analyzed using both quadrupole-time-of-flight (Q-TOF) and triple quadrupole (QQQ) mass spectrometers for comprehensive detection and quantification.

Non-targeted metabolomics analysis was performed using an Agilent 6560 ion mobility quadrupole-time-of-flight (IM-Q-TOF) mass spectrometer (Agilent Technologies, Santa Clara, CA, USA) operated in positive electrospray ionization (ESI) mode. An Agilent Jet Stream (AJS) ESI source was employed, with the following key parameters optimized: dry gas (N_2_) temperature set to 250 °C and flow rate at 8 L/min; nebulizer gas (N_2_) pressure maintained at 35 psi; sheath gas (N_2_) temperature adjusted to 375 °C and flow rate at 11 L/min; capillary voltage fixed at 3500 V; fragmentor voltage at 400 V; and mass scan range set to *m*/*z* 50–1500. Data-dependent tandem mass spectrometry acquisition was conducted in “Auto MS/MS” mode to obtain secondary mass spectra for metabolite annotation.

Targeted metabolomics analysis of the annotated metabolites was conducted using an Agilent 6495 triple quadrupole (QQQ) mass spectrometer (Agilent Technologies, Santa Clara, CA, USA), operated in dynamic multiple reaction monitoring (dMRM) mode. An Agilent Jet Stream (AJS) electrospray ionization (ESI) source was utilized, with the following optimized parameters: dry gas (N_2_) temperature set to 200 °C and flow rate at 14 L/min; nebulizer gas (N_2_) pressure maintained at 35 psi; sheath gas (N_2_) temperature adjusted to 250 °C and flow rate at 11 L/min; capillary voltage fixed at 4000 V; high-pressure radio frequency (RF) lens voltage (RT) at 150 V; and low-pressure RF lens voltage at 60 V. All data were acquired using Agilent MassHunter Workstation software (version matched to Agilent 1290 UHPLC system; Santa Clara, CA, USA). For metabolite quantification, Agilent MassHunter Quantitative Analysis 11.0 software was employed to extract the peak areas from the raw dMRM-acquired data, and the extracted peak areas were used for subsequent quantitative calculations.

### 4.4. Sequence Analysis

For molecular characterization and phylogenetic analysis of tomato (*Solanum lycopersicum*) acyltransferase genes, genomic and amino acid sequences of target genes were retrieved from the Phytozome database (Version 13; https://phytozome.jgi.doe.gov/pz/portal.html, accessed on 20 November 2025). For comparative analysis, genomic and amino acid sequences of rice (*Oryza sativa* cv. ZH11) were acquired from the Rice Resource Center (https://ricerc.sicau.edu.cn, accessed on 14 November 2025)and those of maize (*Zea mays* inbred line B73) were downloaded from MaizeGDB (https://maizegdb.org/, accessed on 7 November 2025)—a curated database dedicated to maize genetics and genomics.

Multiple sequence alignment (MSA) of the deduced amino acid sequences was performed using ClustalW (https://www.genome.jp/tools-bin/clustalw, accessed on 29 November 2025) with default parameters (gap opening penalty: 10; gap extension penalty: 0.2). Phylogenetic analysis was conducted based on the MSA results using the neighbor-joining (NJ) method [44] implemented in MEGA software (version 7.0) [29]. The Poisson model was selected for evolutionary distance calculation, and bootstrap validation with 1000 replicates was performed to evaluate the statistical support for each node in the phylogenetic tree.

### 4.5. RNA-Seq Analysis

Total RNA samples that passed quality control were used for library construction. mRNA was enriched from the total RNA using oligo(dT) magnetic beads and subsequently fragmented under appropriate conditions. First- and second-strand cDNA were then synthesized, and the resulting double-stranded cDNA was subjected to end repair, 3′-adenylation, and adaptor ligation, followed by PCR amplification. The amplified products were denatured into single-stranded DNA and circularized to generate single-stranded circular DNA libraries, while non-circularized linear DNA was removed by digestion. Library quality and fragment size distribution were assessed using an Agilent 2100 Bioanalyzer (Agilent Technologies, Santa Clara, CA, USA). Single-stranded circular DNA molecules were amplified by rolling circle amplification to form DNA nanoballs, which were loaded onto high-density nanoarrays and sequenced using combinatorial Probe-Anchor Synthesis (cPAS) technology. Library preparation was performed using the VAHTS Universal V6 RNA-Seq Library Prep Kit for MGI^®^, and sequencing was carried out on the DNBSEQ T7 platform(MGI Tech Co., Ltd., Shenzhen, China).

The raw sequencing data were processed using Fastp (v0.23.4) with default parameters to remove the adapter sequences and the low-quality sequences in the sequencing data. The quality-controlled paired-end sequencing data were aligned to the corresponding reference genomes (*Zea mays* B73 RefGen_v4, *Oryza sativa* v7.0, *Solanum lycopersicum* SL4.0) using Hisat2 (v2.2.1) with default parameters. Quantification was performed using StringTie (v2.1.4), and the raw quantification results were standardized using TPM (transcripts per million). DEseq2 was used to identify DEGs (differentially expressed genes) among samples. Genes with |log2(Fold Change)| > 1 and *p*-value < 0.05 were considered as DEGs. R software (version 4.3.1) were used for GO functional enrichment and KEGG pathway analysis.

### 4.6. In Vitro Enzyme Activity Assay

The target fragment was cloned into the glutathione (GST)-tagged pGEX-4T-1 expression vector via homologous recombination (Vazyme, Nanjing, China). Methods for recombinant protein analysis and enzyme activity assay were referenced from previous reports, with slight modifications based on the protocol described by Peng [42]. Induction of recombinant protein expression was performed using isopropyl-β-D-thiogalactoside (IPTG) at a final concentration of 0.4 mM, at 16 °C for 16 h. The in vitro activity assay of acyltransferase was conducted in a total volume of 20 μL reaction system, containing 1 mM substrate, 200 mM acyl donor, 2.5 mM MgCl_2_, 500 ng purified protein, and 100 mM Tris-HCl buffer (pH 7.5). After incubating the reaction mixture at 37 °C for 60 min, the reaction was terminated by adding 50 μL of ice-cold methanol. The supernatant was filtered and subjected to LC-MS analysis. Phenolamides were quantified using peak area integration calibrated with authentic samples. The phenolamides generated in the in vitro reaction were identified by comparing their retention times with those of standard substances and through the inference of tandem mass spectra.

### 4.7. Molecular Docking

Molecular docking was conducted using AutoDock (version 1.5.7) software. The structures of small-molecule ligands were retrieved from the PubChem database (https://pubchem.ncbi.nlm.nih.gov/, accessed on 23 November 2025) and preprocessed with AutoDockTools (version 1.5.7). The X-ray crystal structure of the target protein was obtained from the Pfam database (http://pfam.xfam.org/, accessed on 24 November 2025). The docking grid box was centered on the active pocket, with parameters set to default values. For each ligand, 10 docking conformations were generated, and the conformation with the lowest binding energy was selected for subsequent analysis [45]. The protein–ligand interactions were visualized using PyMOL (version 2.3.0).

## Data Availability

The original contributions presented in this study are included in the article. Further inquiries can be directed to the corresponding author.

## Supplementary Materials

The following supporting information can be downloaded at: https://www.mdpi.com/article/10.3390/plants15020299/s1, Supplementary Figure S1. Total ion chromatograms (TIC) of metabolites in different crops and tissues obtained via HPLC-ESI-QTOF-MS/MS analysis. Supplementary Figure S2. Heatmap of metabolite accumulation across different categories among the three crop species. Supplementary Figure S3. Bar chart showing the number of DAMs for different classes of metabolites in maize, rice, and tomato under drought stress. Supplementary Figure S4. Gene–metabolite correlation networks in maize and tomato under drought stress. Supplementary Figure S5. Molecular Docking of Four Proteins (SlPHT3, OsPHT4, ZmPHT4, ZmASFT) with Ligands (Tam, Agm, Put), Respectively. Supplementary Figure S6. Multiple Sequence Alignment of Amino Acid Sequences of Four Proteins and Structural Localization of Active Center Mutations in ZmASFT. Supplementary Figure S7. Comparison of response magnitude and accumulation levels of selected drought-responsive metabolites in maize, rice, and tomato. Supplementary Table S1. Summary of metabolome results. Supplementary Table S2. Summary of differential metabolites. Supplementary Table S3. Significant Pearson correlations (|r| > 0.8, *p* < 0.05) between differential phenolamides and phenylpropanoid pathway genes. Supplementary Table S4. The expression of mRNAs (TPM). Supplementary Table S5. Differentially Expressed Genes (DEGs) under Drought Stress. Supplementary Table S6. Information of all proteins used for phylogenetic tree construction. The protein sequences were retrieved from the corresponding databases, and the phylogenetic tree was constructed using MEGA 7 (neighbor-joining method, 1000 bootstrap replicates, gap deletion: complete deletion). Table S7. Binding Energies Between ligand and target protein from AutoDock Docking. Supplementary Table S8. Primers and gene IDs used in this study. Supplementary Table S9. Homology Analysis with Arabidopsis.
