# Supplementary material for: Metabolic Landscape and Core Regulatory Network of Monocotyledonous and Dicotyledonous Plants in Drought Response Based on Multi-Omics
Source: Plants (Basel). 2026 Jan 19;15(2):299. doi: 10.3390/plants15020299 (PMC12844813; doi:10.3390/plants15020299)
Supplement: Supplementary file 1 [file plants-15-00299-s001.zip › plants-4089397-supplementary.pdf]

Supplementary Figure S1. Total ion chromatograms (TIC) of metabolites in different crops and tissues obtained via HPLC-ESI-QTOF-MS/MS analysis.

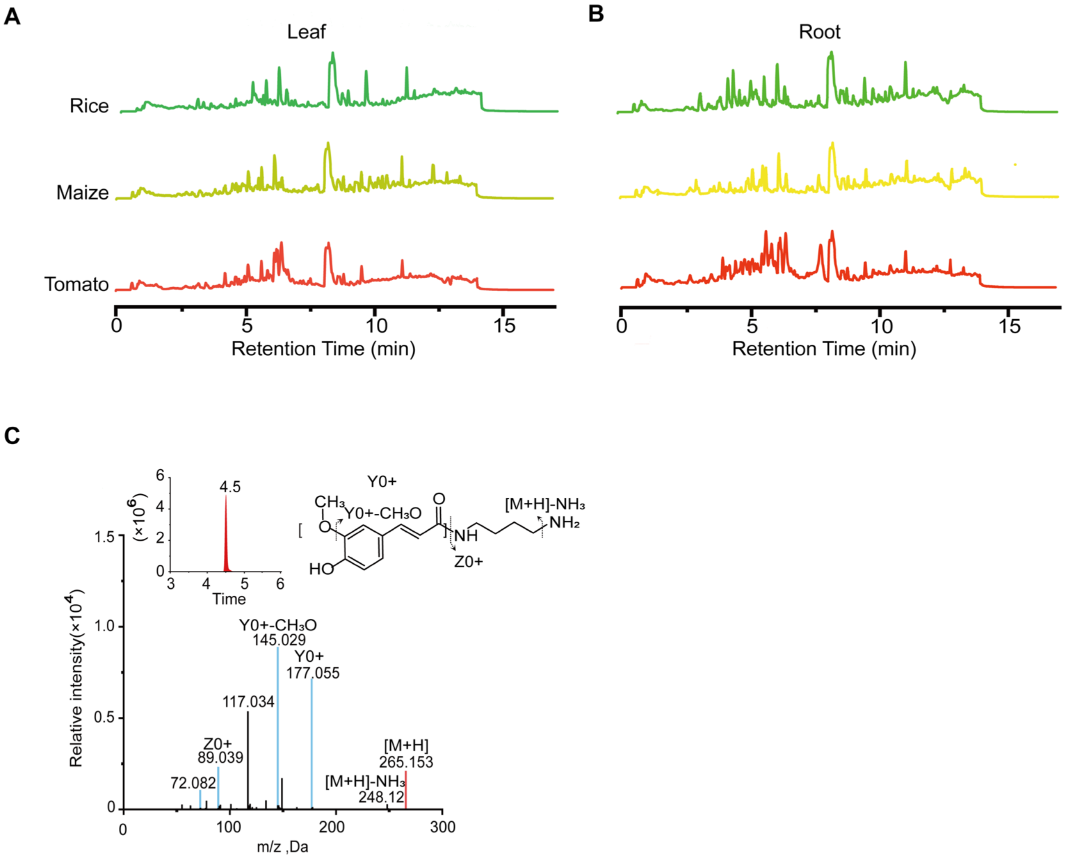

Supplementary Figure S2. Heatmap of metabolite accumulation across different categories among the three crop species.

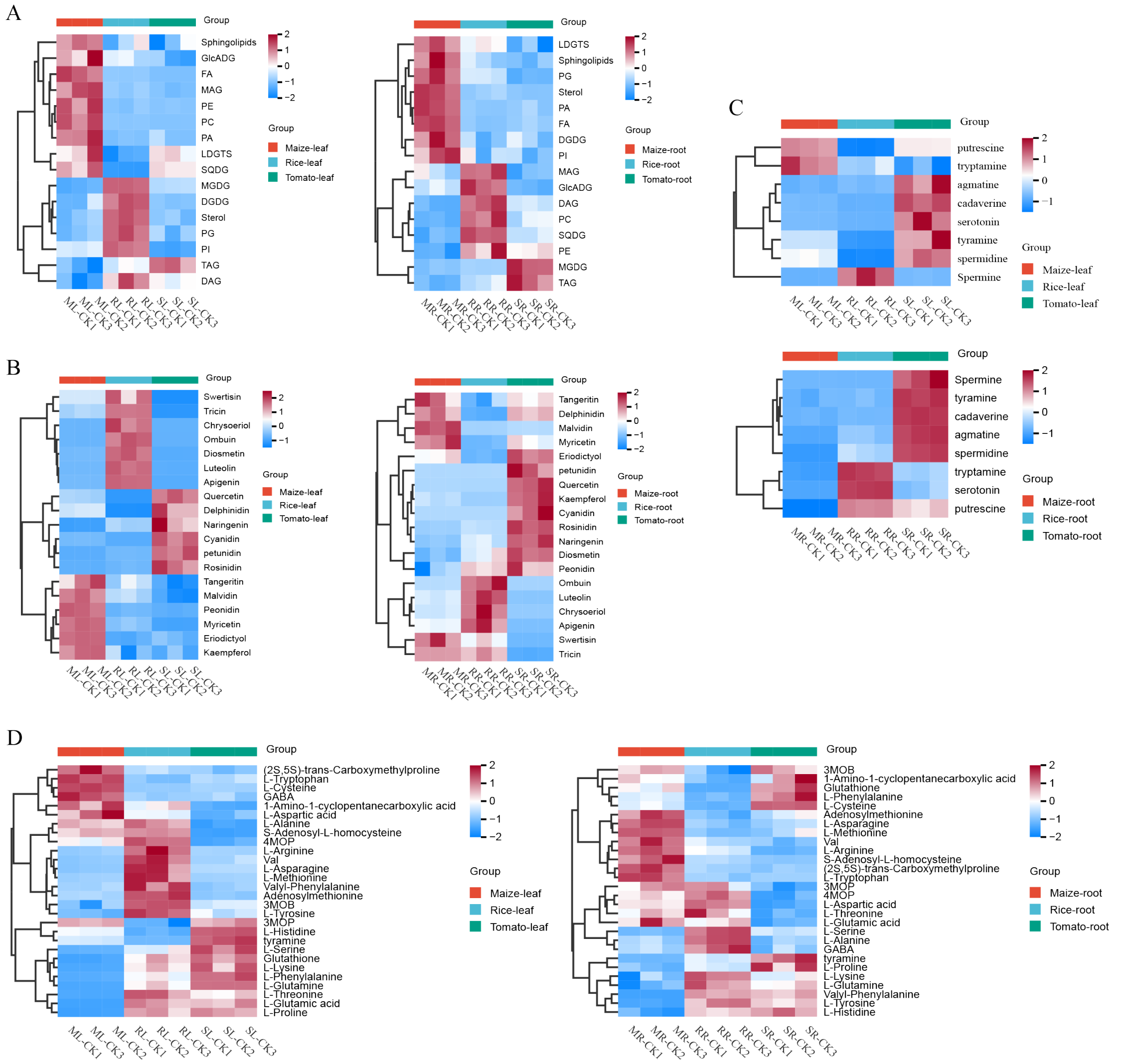

Supplementary Figure S3. Bar chart showing the number of DAMs for different classes of metabolites in maize, rice, and tomato under drought stress.

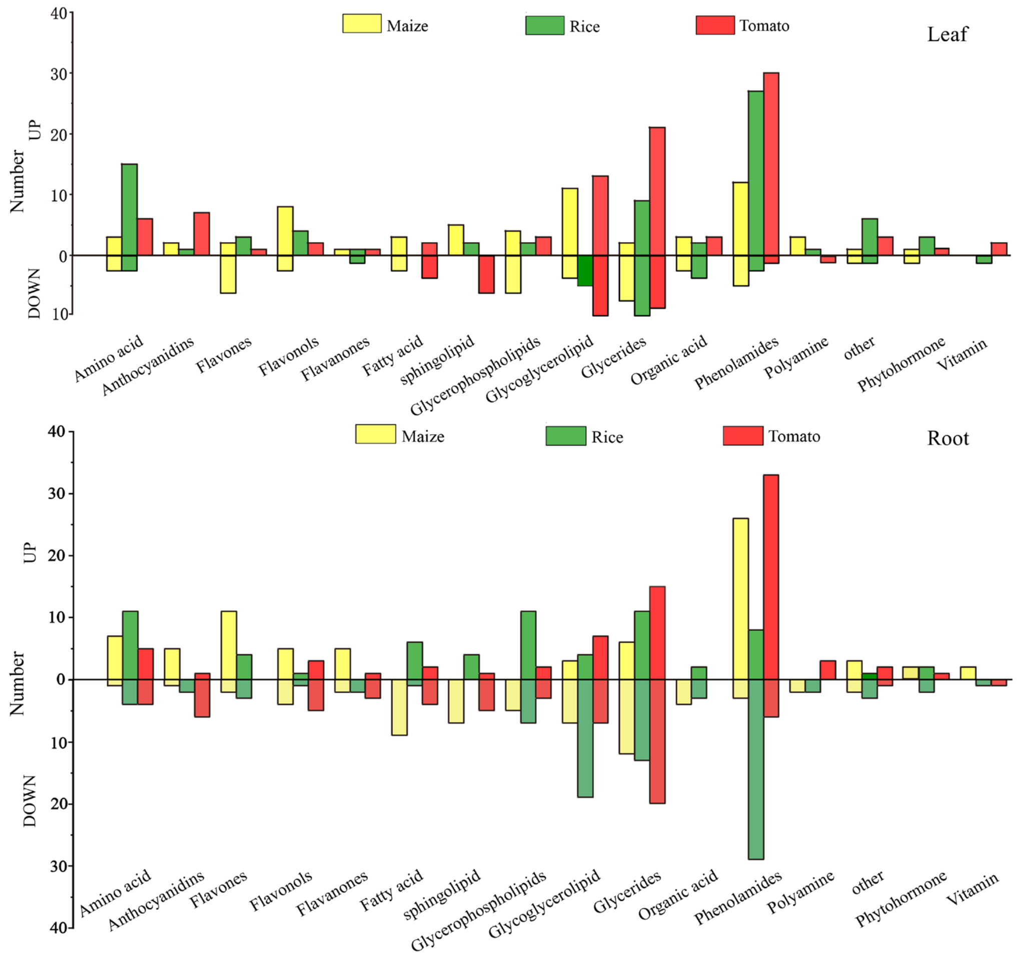

Supplementary Figure S4. Gene-metabolite correlation networks in maize and tomato under drought stress.

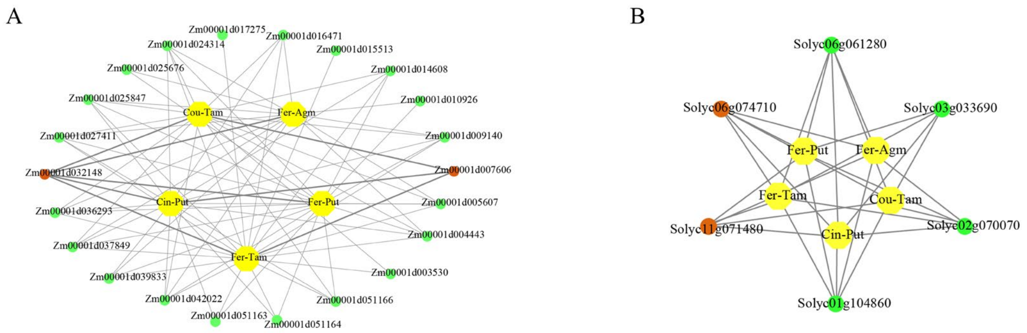

Supplementary Figure S5. Molecular Docking of Four Proteins.

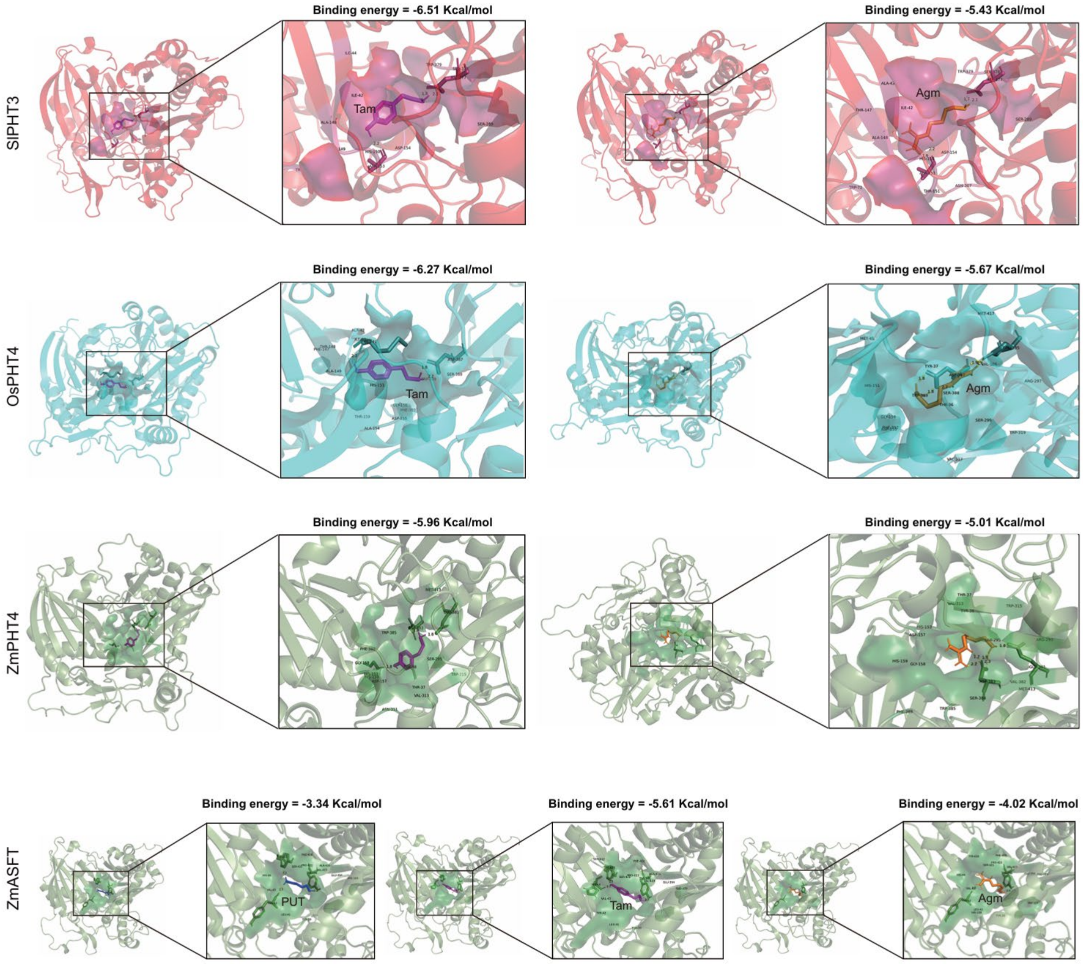

Supplementary Figure S6. Multiple Sequence Alignment of Amino Acid Sequences of Four Proteins and Structural Localiza-tion of Active Center Mutations in ZmASFT . Regions with a red background indicate the highly conserved amino acid residues among the different proteins.

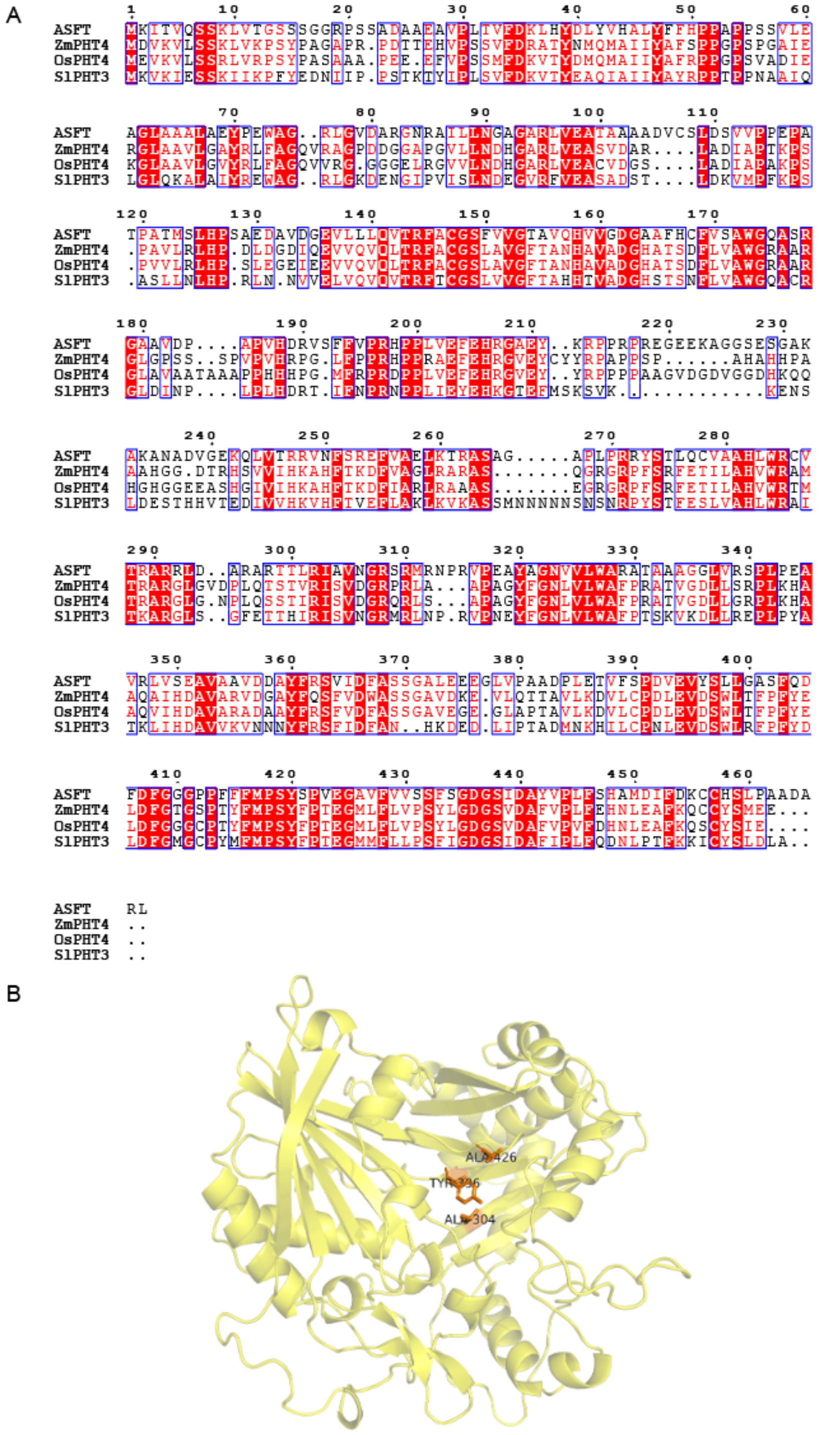

Supplementary Figure S7. Comparison of response magnitude and accumulation levels of selected drought-responsive metabolites in maize, rice, and tomato.

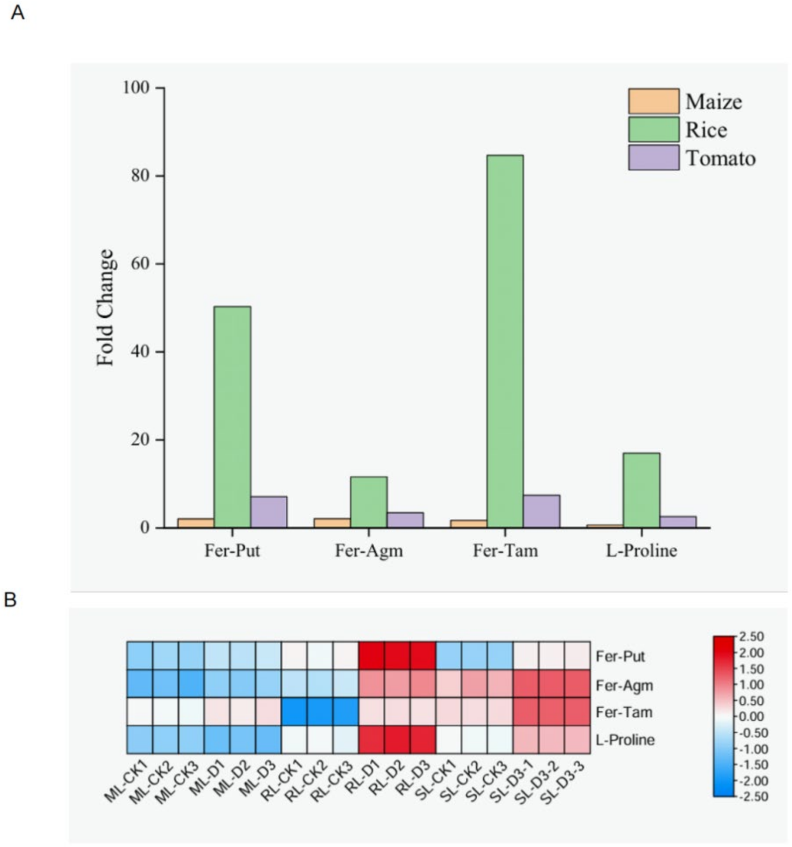

1. Zhu, J.-K. Abiotic Stress Signaling and Responses in Plants. *Cell* **2016**, *167*, 313–324.
2. Sharma, A.; Gupta, A.; Ramakrishnan, M.; Van Ha, C.; Zheng, B.; Bhardwaj, M.; Tran, L.-S.P. Roles of abscisic acid and auxin in plants during drought: A molecular point of view. *Plant Physiol. Biochem.* **2023**, *204*, 108129.
3. Kashyap, A.; Jiménez-Jiménez, Á.L.; Zhang, W.; Capellades, M.; Srinivasan, S.; Laromaine, A.; Serra, O.; Figueras, M.; Rencoret, J.; Gutiérrez, A.; et al. Induced ligno-suberin vascular coating and tyramine-derived hydroxycinnamic acid amides restrict *Ralstonia solanacearum* colonization in resistant tomato. *New Phytol.* **2022**, *234*, 1411–1429.
